# Editorial: Uncovering Drug Resistance During Cancer Therapy

**DOI:** 10.3389/fgene.2022.945842

**Published:** 2022-07-19

**Authors:** Haitao Wang, Rui-Hong Wang, Jian-Guo Zhou, Weilong Hou, Ada Hang-Heng Wong

**Affiliations:** ^1^ Thoracic Surgery Branch, Center for Cancer Research, National Cancer Institute (NIH), Bethesda, MA, United States; ^2^ Department of Oncology, The Second Affiliated Hospital of Zunyi Medical University, Zunyi, China; ^3^ Translational Radiobiology, Department of Radiation Oncology, Universitätsklinikum Erlangen, Erlangen, Germany; ^4^ Department of Radiation Oncology, Universitätsklinikum Erlangen, Erlangen, Germany; ^5^ Comprehensive Cancer Center Erlangen-Europäische Metropolregion Nürnberg (EMN), Erlangen, Germany; ^6^ AW Medical Co Ltd, Macao, Macao SAR, China

**Keywords:** cancer, oncology, cancer therapy, drug resistance, next generation sequencing, biomarkers

Drug resistance is one of the most difficult problems to tackle during cancer therapy because of its multifaceted nature (Vasan, Baselga et al., 2019). Innate drug resistance is generally caused by cancer heterogeneity, and is further complicated by the unique microenvironment and immune landscape of each individual patient. On the other hand, acquired resistance is primarily induced by treatment. The interplay among cancer, its microenvironment and the immune system during the course of treatment unveils the continuous evolution of drug resistance on the temporal scale. Furthermore, the diverse mechanisms of anti-cancer drugs, from targeting cancer proliferation to immune evasion, often trigger drug resistance via distinct courses. Hence, continuous disease monitoring and multi-timepoint sampling became indispensable to interrogate cancer progression and resistance development over the course of treatment.

In this issue, we discussed the molecular mechanisms of drug resistance during cancer therapy. Driver mutations have long been considered the mainstream mechanism of drug resistance development. For instance, the development of *RAS* mutations to constitutively activate the EGFR/RAS pathway is often seen within weeks during anti-EGFR treatment. However, elucidation of point mutations is insufficient to decipher the cancer landscape, let alone the tumor microenvironment. The advent of sequencing technologies enables broader genome coverage and greater depths to uncover genetic mutations other than point mutations, such as indels and copy number changes, while development of novel sequencing methodologies provides additional information on epigenetic modifications, transcriptional regulation, and so on.

For example, Huang et al., described how the membrane transporter *SLC15A4* could serve as a prognostic biomarker for lung adenocarcinoma patients, even though the mutation rate of *SLC15A* family members is relatively low. This underscores how the increased expression of a transmembrane peptide transporter (Song, Hu et al., 2018) that functions in mTOR-dependent autoantibody production (Kobayashi, Shimabukuro-Demoto et al., 2014) could indicate prognosis. While the basal level of *SLC15A4* is prognostic, Wei et al., demonstrated that *MX2* is upregulated with increasing cancer stage and after renal cell carcinoma cells develop resistance against the anti-angiogenic drug Sunitinib. Prognostic biomarkers do not always correlate with tumorigenesis or cancer progression, but might be by-products of these processes. Pretreatment prognosis suggests innate drug resistance, whereas on-treatment prognosis suggests the development of acquired resistance.

Aside from mRNA expression, microRNAs also play important roles in cancer drug resistance (Si, Shen et al., 2019). Tang et al., described how the microRNA processing protein encoded by *hnRNPA2B1* is upregulated in colon cancer to promote cancer progression via the MAPK pathway. Zheng et al., depicted several microRNAs that are positively correlated to *MSM O 1* expression, which is positively correlated to tumor burden and poor prognosis in cervical squamous cell carcinoma. To date, microRNAs contribute to cancer drug resistance mainly through regulation of gene expression. While some microRNAs directly bind to complementary mRNAs to silence gene expression, others cooperate with long non-coding RNAs to fine tune gene expression.

The mechanisms of drug resistance are nonetheless related to the type of anti-cancer drug. While broad-spectrum chemotherapy and targeted therapy inadvertently focus on pathways related to cancer proliferation, sole characterization of cancer cells would leave out important players in the tumor microenvironment that are critical to the development of drug resistance towards immunotherapy. Even though these players may or may not be direct targets of immunotherapy, they contribute to the success of immunotherapy. Wang et al., reviewed various mechanisms of immunotherapy resistance in glioblastoma, such as the infiltration of M2-type tumor-assoicated macrophages (TAMs) and myeloid-derived suppressor cells (MDSCs). TAMs are suggested to promote drug resistance by modulating the cancer stem cell (CSC) population and suppress the immune system via interactions with cytotoxic and regulatory T cells (Petty and Yang 2017). On the other hand, polymorphonuclear MDSCs, which represents 80% of all MDSCs in the majority of cancer types, mainly suppress T cells (Gabrilovich 2017). Hence, cancer evolves to recruit the host immune system to counteract the effects of immunotherapy. Other non-immune players include cancer-associated fibroblasts (CAFs) that secrete growth factors and cytokines to promote tumor growth in breast cancer (Cosentino, Plantamura et al., 2021). Unequivocably, investigation of the tumor microenvironment and the immune-oncological crosstalk is critical to broaden our understanding of the mechanisms of acquired drug resistance.

Therefore, novel sequencing technologies based on single cells and *in situ* hybridization are developed. These technologies provide details to identify and characterize the tumor microenvironment with unprecedented resolution in terms of cell type and spatial localization. Nevertheless, the enhanced resolution also posits new feasibility problems during clinical translation because it is impossible for any physician to interpret an innumerable list of biomarkers to their patients. Hence, dimensionality reduction by the application of ratiometric parameters comes into play. For example, Wang et al., used the ratio of *SIRT4*/*SIRT6* mRNA expression to serve as prognostic biomarkers for serous ovarian cancer. While both SIRT4 and SIRT6 are histone deacetelylases of the sirtuin family, SIRT4 acts as a tumor suppressor to repress glutamine metabolism (Csibi, Fendt et al., 2013; Jeong, Xiao et al., 2013), a key energy source for cancer cells, whereas SIRT6 exhibits context-dependent pleiotropic roles in cancer (Fiorentino, Carafa et al., 2021). The combination of these two biomarkers with potentially contrasting roles but are functionally linked succeeds in dimensionality reduction. Alternatively, artificial intelligence and computational visualization aids in interpreting complex results, but the *in clinico* application of these methods remains afar.

In conclusion, our understanding of the mechanisms of cancer drug resistance has been consistently broadening and deepening by the advancement of novel technologies and methodologies that no longer limits to genetics, but also encompasses epigenetic and transcriptional mechanisms. We foresee that future integration of omic technologies will further expands our understanding of cancer drug resistance.

## References

[B1] Cosentino G. Plantamura I. Tagliabue E. Iorio M. V. Cataldo A. (2021). Breast Cancer Drug Resistance: Overcoming the Challenge by Capitalizing on MicroRNA and Tumor Microenvironment Interplay. Cancers (Basel) 13 (15), 3691. 10.3390/cancers13153691 34359591 PMC8345203

[B2] Csibi A. Fendt S.-M. Li C. Poulogiannis G. Choo A. Y. Chapski D. J. (2013). The mTORC1 Pathway Stimulates Glutamine Metabolism and Cell Proliferation by Repressing SIRT4. Cell 153 (4), 840–854. 10.1016/j.cell.2013.04.023 23663782 PMC3684628

[B3] Fiorentino F. Carafa V. Favale G. Altucci L. Mai A. Rotili D. (2021). The Two-Faced Role of SIRT6 in Cancer. Cancers (Basel) 13 (5), 1156. 10.3390/cancers13051156 33800266 PMC7962659

[B4] Gabrilovich D. I. (2017). Myeloid-Derived Suppressor Cells. Cancer Immunol. Res. 5 (1), 3–8. 10.1158/2326-6066.cir-16-0297 28052991 PMC5426480

[B5] Jeong S. M. Xiao C. Finley L. W. S. Lahusen T. Souza A. L. Pierce K. (2013). SIRT4 Has Tumor-Suppressive Activity and Regulates the Cellular Metabolic Response to DNA Damage by Inhibiting Mitochondrial Glutamine Metabolism. Cancer Cell 23 (4), 450–463. 10.1016/j.ccr.2013.02.024 23562301 PMC3650305

[B6] Kobayashi T. Shimabukuro-Demoto S. Yoshida-Sugitani R. Furuyama-Tanaka K. Karyu H. Sugiura Y. (2014). The Histidine Transporter SLC15A4 Coordinates mTOR-dependent Inflammatory Responses and Pathogenic Antibody Production. Immunity 41 (3), 375–388. 10.1016/j.immuni.2014.08.011 25238095

[B7] Petty A. J. Yang Y. (2017). Tumor-associated Macrophages: Implications in Cancer Immunotherapy. Immunotherapy 9 (3), 289–302. 10.2217/imt-2016-0135 28231720 PMC5619052

[B8] Si W. Shen J. Zheng H. Fan W. (2019). The Role and Mechanisms of Action of microRNAs in Cancer Drug Resistance. Clin. Epigenet 11 (1), 25. 10.1186/s13148-018-0587-8 PMC6371621 30744689

[B9] Song F. Hu Y. Wang Y. Smith D. E. Jiang H. (2018). Functional Characterization of Human Peptide/Histidine Transporter 1 in Stably Transfected MDCK Cells. Mol. Pharm. 15 (2), 385–393. 10.1021/acs.molpharmaceut.7b00728 29224352 PMC5826766

[B10] Vasan N. Baselga J. Hyman D. M. (2019). A View on Drug Resistance in Cancer. Nature 575 (7782), 299–309. 10.1038/s41586-019-1730-1 31723286 PMC8008476

